# The role of plasma-derived small extracellular vesicles in pre-metastatic niche formation through modulation of macrophages in head and neck squamous cell carcinoma

**DOI:** 10.1038/s41416-025-03001-9

**Published:** 2025-05-05

**Authors:** Diana Huber, Tsima Abou Kors, Lutz Schütt, Linda Hofmann, Annika Betzler, Ramin Lotfi, Franziska Oliveri, Sebastian Schmid, Barbara Wollenberg, Thomas K. Hoffmann, Cornelia Brunner, Marie-Nicole Theodoraki

**Affiliations:** 1 Department of Otorhinolaryngology, Head and Neck Surgery, Ulm University Medical Center, Ulm, Germany; 2 Institute of Clinical Transfusion Medicine and Immunogenetics, German Red Cross Blood Transfusion Service, Baden Wuerttemberg-Hessia, Ulm, Germany; 3 Institute for Transfusion Medicine, University Hospital Ulm, Ulm, Germany; 4 Department of Anesthesiology and Intensive Care Medicine, Ulm University Medical Center, Ulm, Germany; 5 Department of Otorhinolaryngology, Head and Neck Surgery, Klinikum rechts der Isar, Technical University Munich, Munich, Germany; 6 Core Facility Immune Monitoring, Medical Faculty, Ulm University, Ulm, Germany

**Keywords:** Oral cancer, Metastasis, Tumour immunology

## Abstract

**Background:**

Metastases are associated with poor survival in head and neck squamous cell carcinoma (HNSCC) patients and tumour-associated macrophages (TAMs) are important drivers in tumour progression and metastasis formation. Small extracellular vesicles (sEVs) are another important factor that contribute to systemic immunosuppression and pre-metastatic niche formation. Here, we investigate the effect of plasma sEVs from HNSCC patients on pre-metastatic niche formation, directly or through modulation of macrophages.

**Methods:**

Primary macrophages were incubated with sEVs from plasma of HNSCC patients or healthy donors (HD). RNA profiles and inflammatory properties of macrophages were evaluated. Direct and indirect effects of sEVs on chemotaxis, T cell activation, proliferation and epithelial-to-mesenchymal transition (EMT) of tumour cells were investigated.

**Results:**

sEVs of HNSCC patients and HD induced different RNA profiles in macrophages. sEVs induced apoptosis and inhibition of T cell activation, while tumour cells were attracted by sEV-treated macrophages, but not sEVs directly. Proliferation was inhibited by both, sEVs and supernatant of EV-treated macrophages in HNSCC. Additionally, EMT in tumour cells was reversed by HNSCC sEV-treated macrophages.

**Conclusion:**

sEVs from plasma of HNSCC patients transformed macrophages into metastasis-promoting TAMs and inhibited anti-tumour T cells, highlighting the potential of sEVs and TAMs as targets for therapeutic approaches.

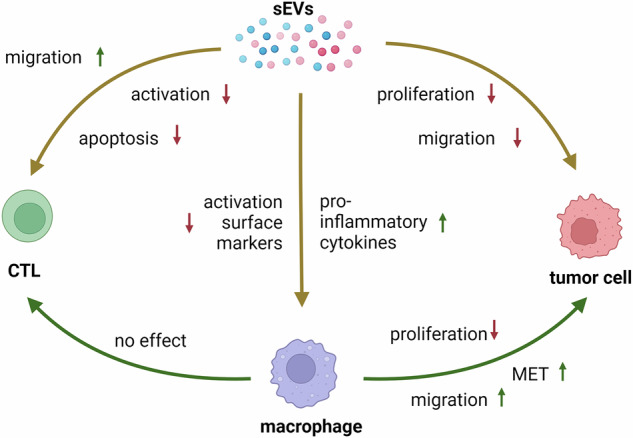

## Introduction

Head and neck squamous cell carcinoma (HNSCC) originate from the mucosal epithelium in the oral cavity, pharynx and larynx and cause the sixth most frequent cases of cancer worldwide [1]. Patients are often diagnosed with advanced stage of the disease and early involvement of lymph node or distant metastases [2], which are also the main cause of cancer-related death [3].

To form a metastasis, tumour cells must complete a cascade of several steps: The initial stage involves the epithelial-to-mesenchymal transition (EMT) of tumour cells, which promotes invasiveness and facilitates detachment from the primary tumour. Next, cells detach from the primary tumour and enter the bloodstream or lymphatic system, through a process known as intravasation, to ultimately reach the site of the future metastasis. While travelling in the circulation, they must evade the immune system’s recognition and elimination. The final stages of metastasis include leaving the circulation and invading into the tissue at the metastatic site, known as extravasation, followed by colonisation of the distant tissue [4–6]. Tumour-associated macrophages (TAMs) were found to promote almost every step in the process of metastasis formation [6]. They induce EMT in primary tumour cells, remodel the extracellular matrix to facilitate dissemination and intravasation into circulation [7] and help tumour cells to survive in circulation [4]. Furthermore, they prepare the microenvironment of the future metastasis, the so called pre-metastatic niche. Before a metastasis occurs, the pre-metastatic niche already acquires cancer-associated properties, like immunosuppression, inflammation and angiogenesis [8, 9]. This leads to recruitment of specific cell types and remodelling of the extracellular matrix, facilitating colony formation for tumour cells. Factors contributing to this pre-conditioning are soluble factors released from tumour cells, like growth factors, cytokines and chemokines. Also, extracellular vesicles (EVs) are very important contributors and are being increasingly investigated regarding their role in pre-metastatic niche formation [9–11].

EVs are produced by every cell type and contain molecules of the parental cell, including proteins, miRNA, mRNA, DNA and lipids. Small EVs (sEVs), earlier called exosomes, have a size of 30–150 nm and are of particular interest because of their origin from the endoplasmic reticulum, where they get packed with specific molecules from the parental cell. Upon release into the extracellular space, they are distributed throughout the whole body, which makes them perfectly suitable as biomarkers for liquid biopsies, including in HNSCC [12–14]. In addition, they can mediate cellular communication by transferring cargo or interacting with the surface receptors of the recipient cell [15]. Cancer cells secrete sEVs that contain immunoregulatory factors and several studies reported systemic immunosuppression in HNSCC patients induced by circulating tumour-derived EVs [12, 16–19]. The immunomodulatory components remain protected from degradation within the EVs and can target cells at distant locations, contributing to the formation of a pre-metastatic niche [20, 21]. Ludwig et al. demonstrated that sEVs from HNSCC cell lines were able to reprogramme macrophages to a tumour-promoting and pro-angiogenic phenotype by modulation of transforming growth factor β (TGF-β)-containing EVs [22]. This pro-tumour effect of HNSCC EVs on macrophages was also confirmed by another study, where exosomes promote malignant migration of oral squamous cell carcinoma by altering macrophage properties [23]. In the context of pre-metastatic niche formation, EVs were shown to recruit bone-marrow derived cells, endothelial progenitors and mesenchymal cells to the niche and contribute to expression of pro-inflammatory cytokines and vascular leakiness [10, 24–26].

To gain further insight into the impact of circulating sEVs on macrophages and their involvement in the formation of pre-metastatic niches, we examined the influence of sEV-treated macrophages on several key processes that are essential for metastasis. This study is the first to investigate how plasma-derived sEVs from HNSCC patients modulate metastasis-promoting properties of primary macrophages in comparison to sEVs derived from the plasma of healthy individuals.

## Methods

### Sample acquisition

Small EVs (sEVs) were isolated from blood plasma of healthy donors (HD) or HNSCC patients, that were newly diagnosed and treatment naïve. Every donor provided informed and written consent. Venous blood was collected in citrate tubes by the Department of Otorhinolaryngology, Head and Neck Surgery Ulm and was centrifuged at 1000 × *g* for 10 min, followed by 2500 × *g* for 10 min to separate plasma which was stored in aliquots at −20 °C.

### sEV isolation by size exclusion chromatography and sEV characterisation

sEVs were isolated by differential centrifugation, ultra-filtration and size exclusion chromatography (SEC), as previously described [27]. Briefly, plasma was sequentially centrifuged at 2000 × *g* for 10 min and at 12,000 × *g* for 30 min. The supernatant was filtered through 0.22 µm syringe-driven filters (Millipore, Burlington, USA) and 1 mL of sample was loaded onto sepharose packed columns (sepharose CL-2B, Cytiva, Uppsala, Sweden). The sEVs were eluted with PBS and fraction #4 was collected. To determine the total sEV protein concentration, Pierce BCA Protein Assay (23225, Thermo Fisher Scientific, Waltham, USA) was used according to the manufacturer’s instructions and the sEVs were concentrated to the desired concentration for further experiments using 100 kDa cutoff centrifugal filters (Millipore, Burlington, USA).

To confirm successful isolation of sEVs, samples were characterised by western blot, nanoparticle tracking analysis and transmission electron microscopy (Supplementary Fig. S1), which is in line with the minimal information for studies of extracellular vesicle (MISEV) 2018 guidelines [28]. These methods are performed routinely in our lab and isolated sEVs can be assigned to EV-TRACK ID: EV200068.

### Cell isolation from buffy coats and macrophage generation

Monocytes and CD8^+^ T cells were isolated from buffy coats from healthy donors provided by the German Red Cross (DRK Ulm). CD8^+^ T cells were isolated by negative selection from Peripheral Blood Mononuclear Cells (PBMCs) using the CD8^+^ T cell isolation Kit (Miltenyi Biotech, Bergisch Gladbach, Germany) according to the manufacturer’s instructions. Purity was determined via flow cytometry with anti-CD8 antibody (BD Biosciences, Franklin Lakes, NJ, USA) after isolation and cells were used for subsequent experiments with a purity of at least 90%.

Monocytes were isolated by CD14 positive selection using CD14 MicroBeads (Miltenyi Biotech) according to the manufacturer’s instructions. 0.5 × 10^6^ cells were seeded into 24-well plates in 1 mL Iscove’s Modified Dulbecco’s Medium (IMDM from Gibco, Waltham, MA, USA) containing 10% exosome-depleted FBS (Gibco), 1% ZellShield (Minerva Biolabs, Berlin, Germany) and 1000 U/mL GM-CSF (Miltenyi Biotech) per well for the differentiation into macrophages. After 3 days, half of the medium was exchanged by IMDM containing 2000 U/mL GM-CSF for another 3 days. On day 6, differentiated macrophages were incubated with 15 µg/mL sEVs in 100 µL PBS or the same volume of PBS as negative control and subsequently analyzed.

### RNA-Sequencing of macrophages

Macrophages were incubated with 15 µg/mL sEVs for 24 h, 3 wells of a 24-well plate with the same conditions were pooled and RNA was isolated using the RNeasy mini-Kit (Qiagen, Hilden, Germany) according to the manufacturer’s instructions. RNA purity was measured using a Nanodrop 1000 and RNA integrity (RIN-) values by TapeStation analysis (Agilent). 200 ng of total RNA, measured by Qubit® analysis was used as input material for library construction using TruSeq RNA Sample Preparation Kit v2 (Illumina). Correct size distribution of the libraries was determined on a DNA1000 ScreenTape® using a TapeStation 4200 (Agilent). Multiplexed libraries were sequenced on an Illumina NextSeq550 using a NextSeq 500/550 High Output Kit v2.5 (75 Cycles) to generate ~30 M of single end 75 base pair reads per library. FASTQ were generated using Illumina bcl2fastq v2.20.0.422. For alignment Bowtie 2 version 2.5.1 was applied with human reference genome GRCh38.p13 (GenBank Assembly ID GCA_000001405.28) using standard settings. The resulting SAM were sorted into BAM by samtools 1.17. Raw read counts for GRCh38 were produced via htseq-count 0.5.4p3-2 using the GTF for GRCh38.p13 (GCA_000001405.28).

### Macrophage polarisation and differentiation markers

To examine the effect of sEVs on macrophage polarisation and activation, macrophages were incubated with sEVs for 48 h. The supernatant was collected to measure cytokine production and cells were labelled with the following antibodies for analysis with the Cytek Aurora Spectral Flow Cytometer (Cytek Biosciences, Amsterdam, Netherlands): CD163 (#12-1639-42) and CD206 (#17-2069-42) were obtained from Invitrogen (Waltham, MA, USA), CD68 (#333806), CD80 (#305236), CD86 (#305423), CD11b (#301356), CD11c (#337218), HLA-DR (#307633), Arginase-1 (#369708), PD-L1 (#329706), CD39 (#328206) from BioLegend (San Diego, USA). For staining of cell surface markers, the cells were detached, washed and blocked with Human TruStain FcX Fc receptor blocking solution (BioLegend), before antibodies were added for 30 min at 4 °C. For intracellular staining of CD68 and Arginase-1, the cells were fixed with fixation buffer (BioLegend) and permeabilized with intracellular staining permeabilization wash buffer (BioLegend) prior to antibody incubation. Furthermore, viability of the cells was assessed by staining non-permeabilized cells with ViaDye Red (Cytek Biosciences, Amsterdam). Secreted cytokines interferon γ, interleukin (IL)-1β, IL-6, IL-10, CC-chemokine ligand (CCL)5, CCL22, C-X-C motif chemokine (CXCL) 10 and tumour necrosis factor (TNF) were analyzed by Multiplex Assay from Merck Milipore using Luminex Technology. To measure TGF-β, samples were activated by incubation with 1 N HCl followed by neutralisation with 1.2 N NaOH/HEPES and measured separately by ELISA (R&D Systems, Minneapolis).

### Tumour cell culture

For functional studies with tumour cells, the HNSCC cell line UD-SCC-1 cells was used, obtained from University of Duesseldorf. The cells were authenticated by STR and tested for mycoplasma regularly and cultivated in Dulbecco’s Modified Eagle’s Medium (DMEM, Gibco) supplemented with 10% Fetal Bovine Serum and 1% ZellShield at 37 °C with 5% CO_2_.

### Tumour cell growth assays

To investigate effects on tumour cell proliferation, 50,000 UD-SCC-1 cells were stained with Cell Trace^TM^ CFSE (Invitrogen), seeded in 12- well plates and incubated with conditioned medium (CM) from macrophages, which were incubated with sEVs for 48 h, or with sEVs directly. CM was diluted 1:3 with fresh medium. After the 48 h or 72 h, respectively, the intensity of the CFSE staining was assessed by flow cytometry. Depicted are the difference of median fluorescence intensities (MFI) of the samples at initial time point and after incubation with sEVs or CM.

To measure tumour cell growth in 3D, spheroids of UD-SCC-1 cells were cultivated by seeding cells in sphericalplate 5D (Kugelmeiers, Erlenbach, Switzerland) in tumorsphere medium (DMEM/F12 containing 20 ng/mL EGF, 10 ng/mL FGF, 5 µg/mL Insulin, 0.4% BSA, B27 supplements and 1% ZellShield). The cells were incubated with either 15 µg/mL sEVs or CM of sEV-treated or untreated macrophages for 24 h and imaged with Axio Observer (Zeiss, Oberkochen, Germany).

### Efferocytosis assay

UD-SCC-1 cells were pre-treated with 1 µM staurosporine for 24 h to induce apoptosis and stained with pHrodo® red cell labelling kit for phagocytosis assays (Sartorius, Stonehouse, UK) according to the manufacturer’s instructions. Macrophages were pre-treated with sEVs for 48 h before labelled apoptotic tumour cells were added for 24 h. After co-incubation, cells were detached, labelled with HLA-DR and EpCAM (130-110-998, Milteniy) antibodies and measured at the Gallios flow cytometer from Beckmann Coulter. The percentage of pHrodo red^+^ macrophages, defined as EpCAM^-^ and HLA-DR^+^, was determined. Since the dye only exhibits a fluorescent signal in acidic milieu, e.g., in phagolysosomes, the signal intensity correlates with the rate of phagocytosis.

### Transwell migration assays and EMT marker expression

UD-SCC-1 cells were seeded on transwell inserts with a pore size of 8 µm (Corning, Glendale, USA). To the lower chamber, either 500 µL of medium containing 15 µg of sEVs or PBS, or CM from sEV-treated and untreated macrophages was added. After 12 h of incubation, non-migrated cells above the filter were removed with cotton swabs. Migrated cells on the bottom side of the filter were fixed with methanol, stained with 0.2% crystal violet in water and imaged at the Axio Observer (Zeiss). The area occupied by stained cells was analyzed using ImageJ.

For T cell migration assays, transwell inserts with a pore size of 5 µm were used. CD8^+^ cells were activated with Immunocult CD3/CD28 T cell activator (Stemcell Technologies, Cologne, Germany) and 10 ng/mL IL-2 for 6 h prior to seeding 200,000 cells per well on transwell inserts. To the lower chamber, 500 µL of CM or sEVs in medium was added, as described above. After 1.5 h of incubation, the filters were removed carefully and the cells in the lower chamber were and counted by flow cytometry (Beckmann Coulter) with a fixed time of acquisition of 1 min.

Furthermore, expression of markers for EMT was assessed after incubation of UD-SCC-1 cells with CM or sEVs for 72 h. The cells were lysed with RLT buffer, RNA was isolated using RNeasy mini-Kit (Qiagen) according to the manufacturer’s instructions and cDNA was synthesised using the QuantiTect Reverse Transcription Kit (Qiagen). qPCR was performed using QuantiNova SYBR Green Kit (Qiagen) with primers obtained from biomers (Ulm, Germany) with 5’-GGAGTCCTGCATACGAG-3’ and 5’-TCTGGAGGACTAGAGG-3’ for TWIST, 5’-AGATGCATATACCCAC-3’ and 5’-CCTCATGTTTAGGAGA-3’ for SLUG, 5’-TCGGAAGCCTCAGCGA-3’ and 5’-AGATGAGCATAGCGAG-3’ for SNAIL and 5’-GACCAGTCAAGGACAT-3’ and 5’-GTGTCAATTAATCAAG-3’ for HPRT.

### T cell activation and apoptosis assay

T cells were isolated and activated for 6 h as described above. After 16 h of co-incubation with sEVs, percentage of CD69^+^ cells was quantified by flow cytometry. For apoptosis assay, T cells were activated for 16 h and co-incubated with sEVs for 6 h, before measuring of Annexin V^+^ and PI^+^ dead cells using the dead cell apoptosis detection kit with Annexin V-FITC from Invitrogen.

### Statistical analysis

The transcriptome data analysis was conducted using R version 4.3.3. The process of data wrangling employed the tidyverse suite (version 2.0.0), data.table (version 1.15.4), and dplyr (version 1.1.4). Differentially expressed genes (DEG) were determined using deseq2 [29] and identified using an absolute log fold change (LFC) greater than 1 and a false discovery rate (FDR) less than 0.05 as thresholds. Gene names were obtained through the biomaRt package (version 2.58.2). KEGG pathway enrichment analysis was performed with the clusterProfiler package (version 4.8.3) and the org.Hs.eg.db database (version 3.17.0), considering pathways with a p-value below 0.05 as significant. Visualisation tasks were carried out using ggplot2 (version 3.3.6).

Data were analyzed using GraphPad Prism 10.2.2. Data were tested for normality and pairwise comparisons were performed with Student’s t-test for parametric and Mann-Whitney U test for non-parametric data. * indicates statistically significant differences with *p* < 0.05 (*), *p* < 0.01 (**), *p* < 0.001 (***), *p* < 0.0001 (****).

## Results

### Clinicopathological parameters of HNSCC patients and healthy donors

Clinical parameters of the donors whose plasma was used for sEV isolation are shown in Table 1. Most HNSCC patients were older than 63 years (62.5%) with a mean of 67 and a range between 40 and 88 years. The control group of HD was younger with only 17.5% over 63 years, a mean of 55 and a range from 34 to 77. Donors were mainly male with 58.8% of HD, and 78% of HNSCC patients. The tumour was mostly located in the oral cavity (41%), followed by the oropharynx (31%) and larynx (19%). 31% of the HNSCC patients were tested positive for Human Papilloma Virus type 16 by PCR, 31% negative and 37% were not tested. Furthermore, most tumours were staged as T3 and T4 (38% and 28%) with lymph node metastases (72%) and 66% were classified as high stage/advanced disease according to Union Internationale Contre le Cancer (UICC) version 8. From the HNSCC patients, 34% were non-smokers, 47% were smokers and 19% quit smoking, while 59% of HD did not smoke. Regarding alcohol consumption, 29% of HDs did not drink alcohol and 65% only occasionally, while 38% of HNSCC patients drink alcohol, 28% drink occasionally and 31% do not drink alcohol. The categorisation of alcohol frequency was the same for HNSCC patients and HD.

**Table 1:** Clinical parameters of HNSCC patients and healthy donors included in our study.

	HNSCC patients (*n* = 32)		healthy donors (*n* = 17)	
	*n*	%	*n*	%
Age > 63	20	62.5	3	17.6
Age < 63	12	37.5	14	82.4
	Range: 40–88		Range: 34–77	
Gender				
male	25	78.1	10	58.8
female	7	21.9	7	41.2
Smoking				
smokers	15	46.9	6	35.3
non-smokers	11	34.4	10	58.8
former smokers	6	18.8	0	0
Alcohol consumption				
yes	12	37.5	0	0
no	10	31.3	5	29.4
occasionally	9	28.1	11	64.7
Primary tumour site				
Oral cavity	13	40.6		
Oropharynx	10	31.3		
Hypopharynx	3	9.4		
Larynx	6	18.8		
HPV status				
positive	10	31.3		
negative	10	31.3		
not tested	12	37.5		
T status				
1	3	9.4		
2	8	25		
3	12	37.5		
4	9	28.1		
Nodal status				
N0	9	28.1		
N+	23	71.9		
UICC stage				
I	3	9.4		
II	8	25		
III	14	43.8		
IV	7	21.9		

### Different RNA profiles of HD sEV-treated and HNSCC sEV-treated macrophages

**Fig. 1:**
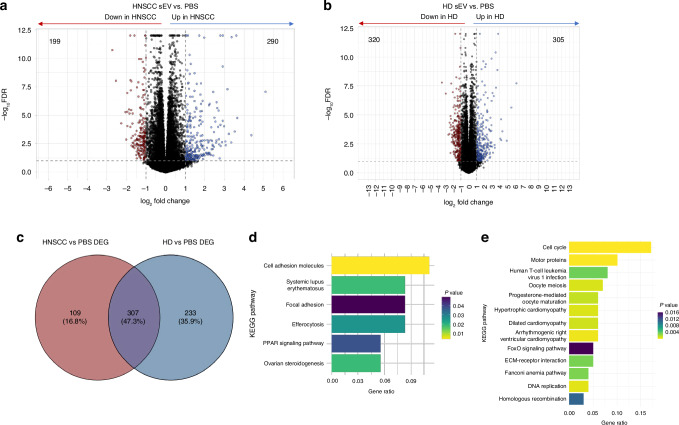
Distinct RNA-profiles of macrophages incubated with HNSCC sEVs or HD sEVs. Volcano plots of differentially expressed genes (DEG) in macrophages incubated with sEVs from plasma of HNSCC patients compared to PBS (**a**) or incubated with sEVs from healthy donors (HD) compared to PBS (**b**). Primary macrophages were generated from monocytes isolated from buffy coats of healthy donors and incubated with 15 µg/mL sEVs of HDs or HNSCC patients for 24 h. **c** Venn diagram of genes differentially regulated by HNSCC sEVs only, HD sEVs only or by both in comparison to PBS. **d** KEGG annotation pathways associated with differentially expressed genes of HNSCC sEVs exclusive (109 genes from **c**). **e** KEGG annotation pathways associated with differentially expressed genes of HD sEVs exclusive (233 genes from **c**). The analysis was performed with macrophages from three different buffy coats and sEVs from 20 HNSCC patients and 8 HDs.

To elucidate the different effects of sEVs on macrophage transcriptomes, macrophages were incubated with plasma-derived sEVs from HDs or HNSCC patients and DEG compared to macrophages incubated with PBS are depicted in Fig. 1.

The only gene downregulated in HNSCC sEV-treated macrophages compared to HD sEV-treated macrophages was FABP4 (fatty acid binding protein 4). When comparing PBS-treated macrophages with HNSCC-sEVs-treated macrophages (Fig. 1a), 199 genes were found to be downregulated in HNSCC-sEV-treated compared to PBS and 290 genes were upregulated. With HD-sEV-treated macrophages, 320 genes were found to be downregulated compared to PBS-treated macrophages and 305 were upregulated (Fig. 1b). Regarding the identity of DEGs, 307 genes were affected by both, HNSCC-sEVs as well as HD-sEVs, but 233 genes were only found to be differentially expressed in macrophages treated with HD-sEVs and 109 genes were only regulated by HNSCC-sEVs (Fig. 1c). KEGG annotation analysis of these 109 HNSCC-sEV-regulated genes revealed association with cell adhesion molecules, focal adhesion, efferocytosis and PPAR signalling pathway, among others (Fig. 1d). In contrast, no cancer related KEGG annotations were found in genes only affected by HD-sEVs (Fig. 1e).

### sEVs induce a mixed phenotype in macrophages

**Fig. 2:**
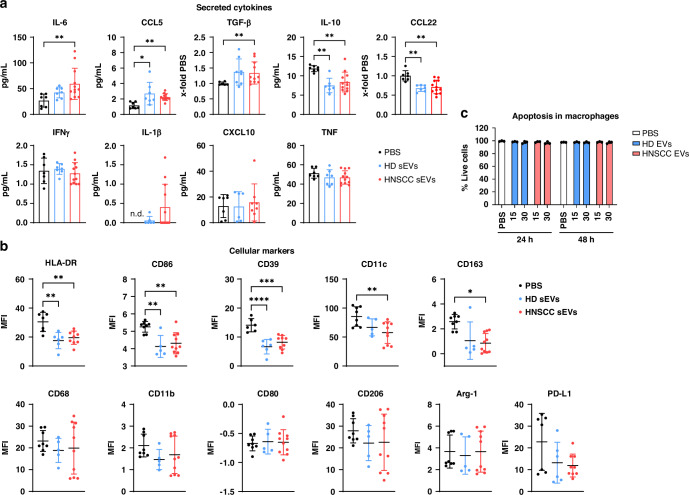
Induction of mixed polarisation phenotype after incubation with HNSCC sEVs. **a** Cytokines measured in the supernatant of macrophages analyzed by Millipore Multiplex Assay with Luminex Technology. Macrophages were incubated with PBS (black), sEVs from plasma of healthy donors (blue) or from HNSCC patients (red) for 48 h. **b** Expression of cell surface markers and two intracellular markers measured by spectral flow cytometry. Cell debris and doublets were excluded, and median fluorescence intensities (MFI) are shown in (**b**). **c** Percentage of cells alive after incubation with PBS or with 15 µg or 30 µg of sEVs. Dots in graphs represent individual donors of sEVs (HD: *n* = 7; HNSCC: *n* = 10) from experiments with macrophages from two different buffy coats (mean and SD).

To further investigate the effect of plasma sEVs on macrophages, cytokines released into the supernatant were measured by Multiplex Assay and surface markers of macrophages were measured by flow cytometry. Production of pro-inflammatory cytokines IL-6, CCL5 and TGF-β (Fig. 2a) were found to be upregulated in macrophages incubated with HNSCC-sEVs, while two anti-inflammatory cytokines IL-10 and CCL22 were significantly downregulated. The latter were also downregulated in macrophages treated with HD-sEVs. While only CCL5 showed an increase by HD-sEVs, no differences were observed in IL-6 and TGF-β. The remaining measured cytokines were not affected by sEV incubation.

Macrophages can be divided into the pro-inflammatory M1 subtype with elevated expression of CD80 and CD86, or into the immunosuppressive M2 subtype, with increased CD206 and CD163 expression. Regarding surface marker expression of macrophages incubated with sEVs, there was a general trend of downregulation of most measured markers (Fig. 2b). HLA-DR, CD86 and CD39 were downregulated significantly after incubation with HNSCC-sEVs and HD-sEVs, while CD11c and CD163 were only downregulated by HNSCC-sEVs. To exclude sEV-mediated apoptosis, cell viability was determined, and no viability decline was visible, even at a higher sEV concentration of 30 µg/mL (Fig. 2c).

Taken together, HNSCC-sEVs downregulated M1 marker CD86, but also M2 marker CD163, as well as other immune relevant markers, indicating a mixed phenotype rather than a clear M1 or M2 polarisation of macrophages.

### sEVs interfere with T cell functionality

**Fig. 3:**
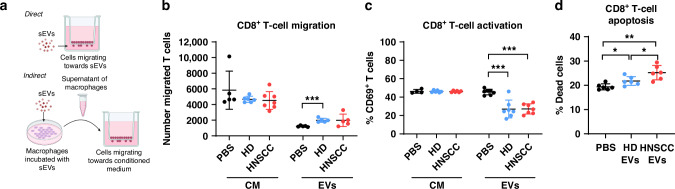
Only direct effect of sEVs induced T cell inhibition and apoptosis. **a** Schematic depiction of chemotaxis directly towards sEVs in medium or indirectly towards conditioned medium (CM) of sEV-treated macrophages. **b** Number of T cells found in the lower chamber of the transwell system, attracted by the corresponding condition of either CM of macrophages incubated with PBS, plasma sEVs from healthy donors (HD) or from HNSCC patients (HNSCC) or with sEVs directly (EVs) were counted using a flow cytometer with 1 min of acquisition. **c** Percentage of activated CD69-expressing T cells after incubation with the respective condition. **d** Percentage of apoptotic T cells, positive for Annexin and PI staining, after incubation with sEVs or PBS. Data points represent individual donors of sEVs (*n* = 7) from experiments with macrophages from two different buffy coats and T cells from three buffy coats (mean and SD).

Since cytotoxic CD8^+^ T cells are main effectors in anti-tumour responses, we also examined the effect of sEVs directly or of CM from sEV-treated macrophages on CD8^+^ T cell chemotaxis, as depicted in Fig. 3a, and on their functionality. Chemotaxis of CD8^+^ T cells was not significantly different between CM from sEV-treated or PBS-treated macrophages (Fig. 3b). However, T cells migrated significantly more towards HD-sEVs directly added to the lower chamber, compared to PBS and a similar trend was observed for the migration towards HNSCC-sEVs. Since CD8^+^ T cells are often exhausted in the tumour microenvironment, we also investigated the effect of sEVs and CM on the activation of CD8^+^ T cells (Fig. 3c) and saw no differences, when CD8^+^ T cells were incubated with CM. When incubating T cells with HD-sEVs as well as HNSCC-sEVs, however, activation was significantly decreased. Furthermore, incubation with HNSCC-sEVs also induced a significantly higher rate of apoptosis in T cells compared to HD-sEVs and PBS (Fig. 3d).

Taken together, these results suggest that CD8^+^ T cells are not modulated by macrophage-derived factors, but by plasma-sEVs directly.

### sEV-treated macrophages prevent tumour cell growth

**Fig. 4:**
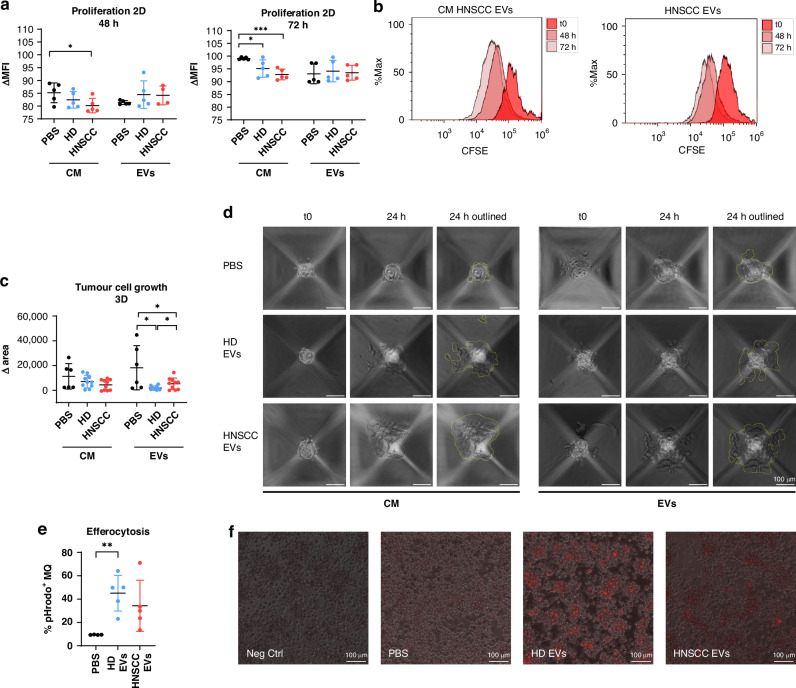
Inhibition of tumour proliferation directly and indirectly. **a** Proliferation of UD-SCC-1 cells in 2D measured by CFSE assay. Cells were incubated with conditioned medium (CM) of sEV-treated macrophages or PBS or with sEVs directly derived from plasma of healthy donors (HD) or HNSCC patients (HNSCC) for 48 h or 72 h. **b** Representative histograms of CFSE intensity before treatment (t0) and after 48 h and 72 h with CM of HNSCC-EV-treated macrophages (left) and HNSCC EVs directly (right). **c**, **d** Proliferation of UD-SCC-1 in 3D spheroids. Cell growth was assessed by calculation of the area occupied by tumour cells before and after 24 h with statistical analysis of the difference in the area (**c**) and representative images of spheroids (**d**). Scale bar = 50 µm. **e**, **f** Efferocytosis assay performed with apoptotic UD-SCC-1 cells. Cells were stained with pHrodo Red, a dye that becomes fluorescent when cells are internalised into acidic phagosomes. The tumour cell-macrophage co-culture was stained with EpCAM and HLA-DR antibodies. For quantification, it was gated on EpCAM^-^ and HLA-DR^+^ macrophages and the percentage of pHrodo Red^+^ macrophages is depicted in (**e**). Representative microscopy images of the co-culture are shown in (**f**). Scale bar = 100 µm. Data points in graphs represent donors of sEVs with mean and SD, *N* = 5.

Proliferation of tumour cells incubated with CM or sEVs was measured after 48 h and 72 h by CFSE staining (Fig. 4a, b). HNSCC-sEVs or HD-sEVs directly added to the tumour cells did not significantly affect proliferation. However, adding CM of HNSCC-sEV-treated macrophages significantly decreased cell growth after 48 h and even more after 72 h of incubation, while CM of HD-sEV-treated macrophages only decreased cell growth after 72 h. Cell growth was also investigated in a 3D model of spheroid cultures of tumour cells with similar results (Fig. 4c, d). Here, tumour cells were cultured to form spheroids that showed an expansion after 24 h, highlighted by the yellow outline. CM of sEV-treated macrophages did not significantly alter cell growth, but incubation with sEVs inhibited spheroid expansion. Tumour cells incubated with HNSCC-sEVs, however, showed a significantly higher expansion compared to HD-sEVs after 24 h.

Efferocytosis is the process of phagocytosis of apoptotic cells by phagocytes, including macrophages. Since efferocytosis was one of the six KEGG annotation pathways associated with DEG of HNSCC-sEVs, we investigated the effect of sEVs on efferocytosis of apoptotic UD-SCC-1 cells by sEV-treated macrophages. The percentage of macrophages, that are pHrodo^+^ was determined by flow cytometry (Fig. 4e). Macrophages pre-treated with HD-sEVs showed remarkably higher rates of efferocytosis with significant difference compared to PBS-treated macrophages. HNSCC-sEVs also increased efferocytosis, but less than HD-sEVs. To confirm the results measured by flow cytometry, Fig. 4f shows representative images of the co-cultured tumour cells and macrophages, with internalised tumour cell components appearing red.

### sEV-treated macrophages attract tumour cells and decrease EMT marker expression

**Fig. 5:**
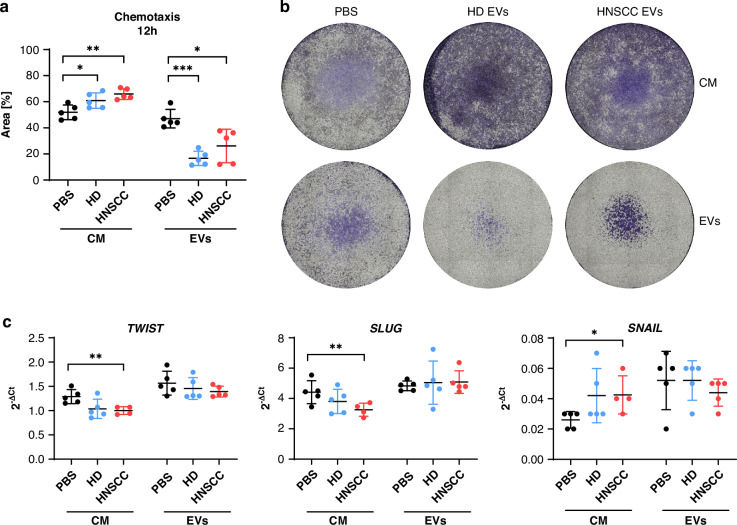
CM of HNSCC-sEV-treated macrophages leads to attraction of tumour cells and reversion of EMT. **a**, **b** Chemotaxis of tumour cells towards CM of sEV-treated macrophages and EVs directly in medium was measured by transwell migration assay after 12 h. Representative membranes of transwell inserts stained with crystal violet are shown in (**b**) and statistical analysis of the occupied area in (**a**). **c** RNA expression of EMT markers *TWIST, SNAIL* and *SLUG* measured by qPCR after incubation with CM of sEV-treated macrophages or EVs directly after 72 h. Data points represent sEV donors with mean and SD, *N* = 5 and CM were collected from macrophages from two different buffy coats.

Since migratory potential of tumour cells is very important in forming metastases, we investigated transwell migration and EMT marker expression of tumour cells. Tumour cell chemotaxis was significantly increased towards CM of sEV-treated macrophages compared to PBS-treated macrophages, with slightly higher migration towards CM of HNSCC-sEV-treated macrophages (Fig. 5a) as observed by a higher coverage of the bottom side of the filter inserts after migration (Fig. 5b). In contrast, chemotaxis was significantly decreased towards HNSCC-sEVs and HD-sEVs directly, compared to PBS. Expression levels of EMT markers *TWIST*, *SNAIL* and *SLUG*, quantified by qPCR, were not significantly affected by adding HNSCC- or HD-sEVs (Fig. 5c). But incubation with CM of HNSCC-sEV-treated macrophages significantly decreased expression of *TWIST* and *SNAIL*, and increased expression of *SLUG* compared to CM from PBS-treated macrophages.

## Discussion

TAMs are important drivers of tumour progression and metastasis and their presence is associated with poor survival in HNSCC patients [30]. They participate in every step of metastasis formation from induction of invasiveness to preparation of the pre-metastatic niche and therefore make a suitable target for anti-cancer therapies preventing metastasis formation. Another crucial factor modulating the pre-metastatic niche are sEVs, which regulate several pivotal biological processes. The objective of this study was to determine whether plasma derived sEVs of HNSCC patients, can transform healthy macrophages into tumour-promoting TAMs, to support growth of secondary tumours in the periphery and to what extent these effects are directly modulated by sEVs.

The inherent biological variability of macrophage donors made it challenging to detect subtle differences induced by sEVs from different sources. Nevertheless, we chose to work with primary macrophages, because they better reflect the in vivo situation, including interindividual variations. It is also important to note that the concentration of plasma sEVs is higher in HNSCC patients than in HD [16]. Given that the concentration of all sEVs was normalised to 15 µg/mL in all experiments, the effect of HNSCC sEVs is likely to be underestimated. Despite small differences between effects of HD- and HNSCC-sEVs, the RNA profiles of macrophages differed depending on the source of sEVs and different processes were targeted by HNSCC compared to HD-sEVs. Only genes differentially regulated by HNSCC-sEVs could be attributed to the KEGG pathways “Cell adhesion molecules”, “Efferocytosis” and “PPAR signalling pathway”. Peroxisome proliferator-activated receptor (PPARs) are nuclear receptors that regulate transcription of genes related to lipid metabolism and immune functions [31, 32]. In the context of cancer, PPAR agonists and antagonists are tested in clinical trials for treatment of several cancer types, providing promising results, by either targeting tumour cells or the associated immune cells [33, 34]. Interestingly, PPARs are activated by fatty acids, which can also be found in exosomes [35]. Even more, exosomes derived from cancer cells were shown to contain higher levels of fatty acids compared to non-cancerous cells and could induce PPARα activation in recipient dendritic cells, leading to immune evasion [36]. Since our results also pointed to a tumour-dependent regulation of PPAR signalling by sEVs, PPAR makes a promising target for future therapeutic approaches.

Another interesting KEGG annotation that was only associated with HNSCC-sEV treated macrophages is efferocytosis. Efferocytosis, the removal of dead cells by phagocytes, is crucial for a healthy microenvironment, because components of dying cells are removed, which can be harmful for surrounding cells and cause secondary necrosis [37, 38]. The fact that efferocytosis prevents an inflammatory reaction also helps tumour cells to evade recognition by the immune system in the TME. On the other hand, removal of tumour cells by phagocytosis is the main anti-tumour function of macrophages [39]. The increased rate of efferocytosis observed in our data indicates a type of activation by sEVs, leading to increased phagocytosis. However, whether this phagocytosis is beneficial for the colonising tumour cells or leads to their removal must be determined.

Macrophages were often described to display immunosuppressive M2-like properties in the TME and contribute to tumour progression. However, recent findings have challenged the traditional theory of M1- and M2-type macrophages, proposing a more complex and continuous spectrum of macrophage subtypes with high plasticity [40]. Even more, Chen et al. reported that EVs from oropharyngeal squamous cell carcinoma cells were able to induce a M1-like phenotype in macrophages that also exhibit pro-tumour properties [23]. The results of our experiments indicated no distinct M1 or M2 polarisation of macrophages incubated with HNSCC-sEVs. Instead, a mixed phenotype was observed, with both M1 and M2 markers being downregulated. The same was observed by Ludwig et al. where no distinct M1 or M2 polarisation was observed after incubation of macrophages with EVs from HNSCC cell lines [22].

Furthermore, anti-inflammatory cytokines CCL22 and IL-10 were found to be downregulated, while pro-inflammatory cytokines were upregulated by incubation with HNSCC-sEVs. Together with the increased levels of efferocytosis, this suggests a pro-inflammatory phenotype of sEV-treated macrophages. At low levels, chronic inflammation is pro-tumorigenic in early stages of tumour development and provides a growth-promoting environment. However, since HD-sEVs induced similar phenotypes, another effect of sEVs could be the activation of macrophages to induce an anti-tumour response. Indeed, Tkach et al. demonstrated that EVs from triple negative breast cancer induced a pro-inflammatory macrophage phenotype, which was even associated with better clinical outcome [41]. Macrophages often have anti-tumour properties during early stages of cancer development, before they transform into tumour-promoting TAMs [42, 43]. Since we use monocyte-derived macrophages from healthy donors in our experiments, macrophages were not exposed to tumour factors in advance and could therefore be assigned to early stages of tumour development. The transformation to tumour-promoting TAMs is often dependent on other cues in the TME, especially hypoxia, which was not present in our experimental set-up, suggesting a dual role of sEV-primed macrophages in tumour initialisation and supporting the theory of highly plastic TAM subtypes. Besides sEVs directly altering the functions of macrophages, sEV-treated macrophages can also affect other cell types.

CD8^+^ T cells did not change their properties when incubated with CM of sEV-treated macrophages, but were mainly affected by sEVs directly, which suppressed activation of T cells and increased apoptosis. This is in line with previous experiments, where not only plasma-sEVs were able to induce apoptosis in CD8^+^ T cells, but also sEVs derived from saliva of HNSCC patients [12]. In our experiments, T cells were only incubated with CM of macrophages, containing soluble factors secreted by macrophages after incubation with sEVs. Effects that are mediated through direct cell-cell contact, like binding cell surface receptors, were not investigated.

In contrast, macrophages were able to interfere with several properties of tumour cells. TAMs were reported to promote cancer cell proliferation in various ways, but in this study, HNSCC sEV-treated macrophages did not promote cancer cell proliferation, neither in 2D nor in 3D models. They even decreased cell growth significantly, suggesting that this typical aspect of TAMs could not be induced by plasma-sEVs of HNSCC patients. As mentioned above, during early stages of metastasis formation, macrophages were described as protective against cancer progression [42, 43], while in later stages tumour-promoting macrophages gradually dominate [42, 44]. Furthermore, because of the high plasticity of macrophages in response to different stimuli, they can generate distinct TAM subsets with complementing abilities to support or prevent tumour growth and metastasis [45–47].

Additionally, this study shows that tumour cells are attracted to a greater extent by CM of HNSCC-sEV-treated macrophages in comparison to untreated and HD-sEV-treated macrophages. EV-dependent modulation of macrophages was essential for this outcome, as tumour cells were not attracted by sEVs directly. In line with this observation, macrophages were described to promote extravasation of tumour cells from the circulation to the pre-metastatic niche by remodelling the ECM and leaving tracks for cancer cells to follow [48]. Also, downregulation of EMT markers by tumour cells was only observed with CM of macrophages incubated with HNSCC sEVs, not with CM of differently treated macrophages or with sEVs directly. EMT is an important process to enable tumour cells to leave the primary tumour. Nevertheless, when tumour cells establish new metastases, the capacity to form cell clusters must be reestablished through the reversal of EMT. The process of mesenchymal-to-epithelial-transition (MET), particularly through the downregulation of Twist, has been demonstrated to be a pivotal step in the formation of metastases and tumour cell proliferation in squamous cell carcinoma [49]. Furthermore, in mouse breast cancer models, myeloid cells in the pre-metastatic lung were responsible for ECM remodelling and induction of MET in metastatic tumour cells [50]. In summary, our results suggest that HNSCC-sEVs primed macrophages to initially attract tumour cells to the pre-metastatic niche and subsequently induce MET, enabling the tumour cells to stay and colonise the distant tissue. At this point, sEV-treated macrophages might exhibit tumour-promoting activities, like chemotaxis of tumour cells and MET induction, but at the same time still have anti-tumour properties, like inhibition of tumour cell growth.

The observed effects of HD-sEVs and HNSCC-sEVs on macrophages were often comparable in our experiments. Since plasma sEVs not only constitute of tumour-derived sEVs but display a mixture of sEVs produced by many different cell types, the effects of sEVs on macrophages in the periphery are similarly complex. Moreover, the impact of sEVs on macrophages represents only one facet within the broader context of a pre-metastatic niche and other important influences, including nutrient deprivation or hypoxia, also play an important role. This fact renders the discovery of several crucial processes regulated by sEVs from HNSCC patients’ plasma particularly intriguing. Especially tumour cell properties were found to be differentially regulated by macrophages treated with sEVs from HD or HNSCC patients. Chemotaxis was increased by HNSCC-sEVs, while EMT marker expression was only significantly altered by HNSCC-sEVs, suggesting a more effective attraction by HNSCC-sEV-treated macrophages and improved colonisation of tumour cells in the pre-metastatic niche. Furthermore, the size of spheroids was significantly increased by HNSCC-sEVs compared to HD-sEVs, indicating a beneficial role of HNSCC-sEVs by promoting tumour growth and by reducing functionality and viability of anti-tumour CD8( + ) T cells. HNSCC-sEVs and macrophages complement each other in directing tumour cells to the pre-metastatic niche and promoting cell growth, resulting in an overall phenotype that facilitates metastasis.

In conclusion, given the high abundance of TAMs in the TME and their involvement in most tumour-progressive processes, targeting TAMs in cancer therapy seems very attractive. However, their high plasticity makes it difficult to effectively target them in therapeutic interventions. Adding to this, we showed that healthy macrophages can be transformed into tumour-promoting macrophages by interaction with circulating sEVs from HNSCC patients. This must be considered for TAM-targeting strategies and makes complete removal of all types of macrophages an attractive approach. However, anti-tumour properties of macrophages, which are also important in eliminating tumour cells, must be preserved. Furthermore, immunosuppressive functions of sEVs are not only observed in the context of macrophages, but also in the context of CD8^+^ T cell inhibition. A combination of targeting approaches combining apoptosis induction in tumour cells, transformation or elimination of TAMs or other immune cells and reduction or modulation of tumour-derived sEVs would therefore be a promising strategy.

## Supplementary information

Supplementary Figure S1

## Data Availability

The datasets generated during this study are available in this manuscript or deposited on Sequence Read Archive (SRA) from NCBI under the accession number PRJNA1215959.
