# Supplementary material for: The role of plasma-derived small extracellular vesicles in pre-metastatic niche formation through modulation of macrophages in head and neck squamous cell carcinoma
Source: Br J Cancer. 2025 May 5;133(1):121–30. doi: 10.1038/s41416-025-03001-9 (PMC12238395; doi:10.1038/s41416-025-03001-9)
Supplement: Supplementary file 1 — Supplementary Figure S1 [file 41416_2025_3001_MOESM1_ESM.pptx]

## Slide 1
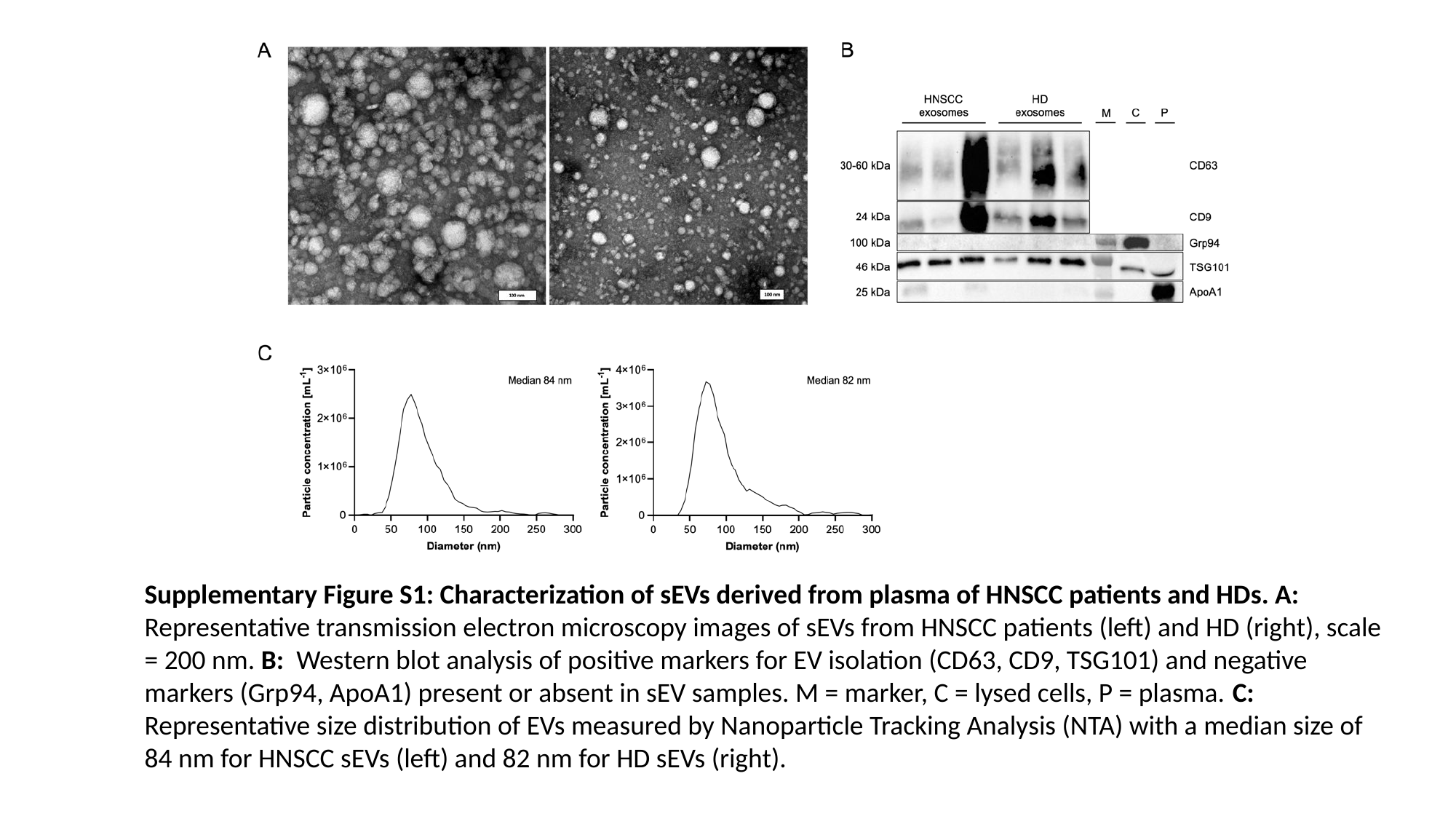

Supplementary Figure S1: Characterization of sEVs derived from plasma of HNSCC patients and HDs. A: Representative transmission electron microscopy images of sEVs from HNSCC patients (left) and HD (right), scale = 200 nm. B: Western blot analysis of positive markers for EV isolation (CD63, CD9, TSG101) and negative markers (Grp94, ApoA1) present or absent in sEV samples. M = marker, C = lysed cells, P = plasma. C: Representative size distribution of EVs measured by Nanoparticle Tracking Analysis (NTA) with a median size of 84 nm for HNSCC sEVs (left) and 82 nm for HD sEVs (right).
